# Predictors of remission in rheumatoid arthritis patients treated with biologics: a systematic review and meta-analysis

**DOI:** 10.1007/s10067-022-06307-8

**Published:** 2022-08-16

**Authors:** Yasmin Khader, Azizullah Beran, Sami Ghazaleh, Wade Lee-Smith, Nezam Altorok

**Affiliations:** 1 Department of Internal Medicine, University of Toledo, 2100 West Central Ave, Toledo, OH 43606 USA; 2 University of Toledo Libraries, University of Toledo, Toledo, OH USA; 3 Department of Rheumatology, University of Toledo, Toledo, OH USA

**Keywords:** Biologic therapy, Rheumatoid arthritis, Disease activity score, Remission

## Abstract

Biologics have emerged as an effective treatment of rheumatoid arthritis (RA). However, there is a significant proportion of patients who fail to respond to biologics. Identifying the predictors that affect the response to biologics remains challenging. A comprehensive literature search of PubMed, Embase, and Web of Science databases was conducted through May 1, 2022. We included all studies that used a multivariate model to assess for the predictors of remission in RA patients treated with biologics. We calculated pooled odds ratios (OR) with 95% confidence intervals (CI) for risk factors reported in ≥ 3 studies using a random-effects model. A total of 16,934 patients with RA who were treated with biologics were included in twenty-one studies. Our study showed that old age (OR 0.98 (0.97, 0.99), *P* < 0.00001), female gender (OR 0.66 (0.56, 0.77), *P* < 0.00001), smoking history (OR 0.86 (0.75, 0.99), *P* 0.04), obesity (OR 0.95 (0.91, 0.99), *P* 0.02), poor functional status (OR 0.62 (0.48, 1.27), *P* < 0.00001), high disease activity (OR 0.90 (0.85, 0.96), *P* 0.0005), and elevated erythrocyte sedimentation rate (OR 0.99 (0.98, 1.00), *P* 0.009) were poor predictors of remission. On the other hand, positive anti-citrullinated protein antibodies (OR 2.52 (1.53, 4.12), *P* 0.0003) was associated with high remission rate. Old age, female gender, obesity, smoking history, poor functional status, high disease activity, and elevated ESR at the time of diagnosis have been associated with poor response to biologics. Our findings could help establish a risk stratification model for predicting the remission rate in RA patients receiving biologics.

**Supplementary Information:**

The online version contains supplementary material available at 10.1007/s10067-022-06307-8.

## Introduction

Rheumatoid arthritis is a chronic autoimmune disease characterized by inflammatory polyarthritis that mainly affects the small joints [1]. Biological disease-modifying antirheumatic drugs (bDMARDs) have emerged as an important advancement in the treatment of rheumatoid arthritis [2]. There are several types of biologics, each of which targets a specific type of molecule involved in the pathogenesis of the disease. These include tumor necrosis factor alpha (TNF-α) inhibitors, such as etanercept, adalimumab, infliximab, certolizumab pegol, and golimumab. Other biologics that target other molecules include abatacept (a selective co-stimulation modulator that inhibits T-cells), rituximab (B-cell inhibitor), tocilizumab (IL-6 receptor antagonist), and anakinra (IL-1 receptor antagonist).

Despite the increasing number of biologics, the ability to achieve complete remission in certain RA patients remains challenging. Approximately 66% of RA patients failed treatment with TNF inhibitors in 6 months of follow-up [3], and a minimum of 10% who tried a second bDMARD had their medication stopped due to lack of response [2]. This suggests that there is a significant proportion of patients who do not respond to bDMARDs.

Several observational studies have identified different predictors of remission in RA patients receiving biologics [4–24]. However, many of these predictors remain inconsistent. Some studies showed that old age, female gender, smoking history, obesity, presence of comorbidities, increased disease activity at the time of diagnosis, increased disease duration, and poor functional status at baseline have been associated with a lower response rate to biologics [4–6, 8, 9, 14, 15, 17]. While other studies showed no significant association between age, gender, and remission rate [15, 18, 23]. Patients with elevated ESR at the time of diagnosis have also shown poor response to biologics in some studies [9, 14], but there was no significant association in other studies [8, 17]. A meta-analysis was also conducted in 2018 to assess for the predictors of remission in RA patients regardless of the treatment that the patients received [4]. In this study, we conducted a systematic review and meta-analysis to assess the strength of association between these predictors and the rate of remission in RA patients treated with bDMARDs.

## Methods

We conducted this systematic review and meta-analysis based on the guidelines of the Preferred Reporting Items for Systematic Reviews and Meta-analysis [5], and Meta-analysis of Observational Studies in Epidemiology [6].

### Data sources and search strategy

We performed a comprehensive search for published studies indexed in PubMed, Embase, and Web of Science databases from inception through May 1, 2022. We also performed a manual search for additional relevant studies using references of the included articles. The following search terms were used: “biologics OR Etanercept OR Infliximab OR Adalimumab OR Certolizumab OR Golimumab OR Anakinra OR Tocilizumab OR Sarilumab OR Abatacept OR Rituximab” AND “relapse OR remission” AND “arthritis OR rheumatoid OR rheumatoid arthritis” AND “risk factors OR predictors.” The search was not limited by language, study design, or country of origin. Two investigators (YK and AB) independently performed the literature search, screened using a priori criteria, and shortlisted the studies for final review. The bibliographic software EndNote was used for screening. Any discrepancies were resolved by a third reviewer (SG).

### Inclusion and exclusion criteria

Studies meeting the following inclusion criteria were included: (1) full-text peer-reviewed publications of retrospective or prospective, cohort or case–control studies, (2) assessed for predictors to response to different types of biologics in RA patients, and (4) reported odds ratio (OR) for this association after multivariate analysis and adjustment of potential confounding factors. We excluded conference abstracts. We also excluded studies reported data based on hazard ratio or univariate analysis rather than multivariate analysis.

### Data extraction

The following data were extracted from the studies: study characteristics (author, publication year, study design, country of origin, and study population), patients’ baseline characteristics, the follow-up duration, and variables that were adjusted in a multivariable analysis. Risk factors that were assessed in at least three studies were included in the meta-analyses. Two investigators (YK and AB) independently extracted the data from the articles, and discrepancies were resolved by a third reviewer (SG).

### Statistical analysis

We performed a meta-analysis of the included studies using Review Manager 5.3 (Cochrane Collaboration, Copenhagen) and Comprehensive Meta-Analysis 3.3 software (Biostat, Englewood, USA). Multivariate adjusted odds ratios (OR) for individual studies were pooled using a random-effects model and reported using a 95% confidence interval (CI) for each risk factor where applicable. Pooling was undertaken if at least three studies reported an odds ratio for a given risk factor. A *P* value < 0.05 was considered statistically significant. Heterogeneity was assessed using the Higgins *I*^2^ index, where *I*^2^ values > 50% implied the presence of significant heterogeneity [7].

### Sensitivity analysis

To evaluate the robustness of results, leave-one-out analysis was attempted for risk factors reported by ten or more studies.

### Bias assessment

We assessed the quality of the included studies using the Newcastle–Ottawa Scale [8]. Two authors (YK and AB) independently assessed each study for bias. For risk factors reported by ten or more studies, publication bias assessment across studies was performed qualitatively by visualization of the funnel plot [9] and quantitatively, using Egger’s regression analysis [10]. A *P* value was generated using Egger’s analysis, and a value of < 0.05 was associated with significant publication bias. If bias was present on Egger’s test, further statistics using the Fail-Safe N test and Duval and Tweedie’s “Trim and Fill” test were used to ascertain the impact of the bias.

## Results

### Study selection

**Fig. 1:**
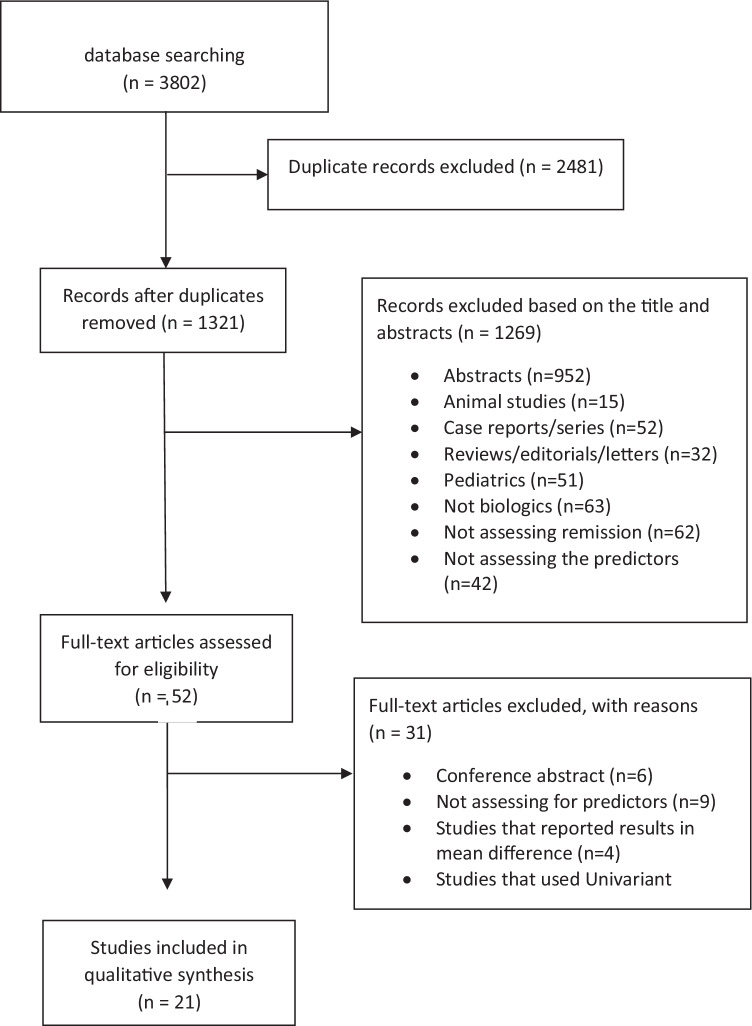
PRISMA flow diagram for the selection of studies

We included a total of 3802 studies in our analysis (647 studies from PubMed, 2076 studies from Embase, 347 studies from Cochrane, and 732 studies from Web of Science). A total of 2481 duplicated studies were excluded, and a total of 1321 studies were reviewed based on the abstracts. Out of these, 1269 studies were excluded after reviewing the title and the abstract. Then, 52 studies were reviewed based on the full text. Thirty-one studies were excluded (nine studies did not assess predictors of remission, four studies reported results in mean difference, twelve studies did not report risk factors that underwent multivariate analysis, and six studies were conference abstracts). Finally, a total of 21 studies [11–31] met our inclusion criteria and were included in our analysis. A PRISMA flowchart that demonstrates how the included studies were selected is shown in Fig. 1.

### Characteristics and quality of included studies

Table 1 shows the characteristics of the studies included in the meta-analysis. All the included studies were published between April 2006 and August 2021. Based on country of origin, six studies originated from Japan [22, 25–29], two studies originated from the USA [14, 24], two studies originated from Italy [15, 20], two studies originated from United Kingdom [11, 21], two studies from France [18, 19], one study from Canada [16], one study from Greece [17], one study from Germany [12], one study from Ireland [30], one study from Australia [31], and one study from Taiwan [23]. Regarding the study design, all the included studies were either retrospective or prospective cohort except of Listing et al. [12] that was a randomized control trial.

**Table 1:** Characteristics of studies included in the meta-analysis

Study/year	Country	Study design	Sample size, (female), *n*	Age, mean (SD), years	Biologics used	Follow-up period (months)	Remission criteria	Variables adjusted in meta-analysis
Atzeni 2014	Italy	Prospective cohort	1300 (1064)	54.6 (13.7)	INF, ETA, ADA	12	DAS28 ≤ 2.6	Age, gender
Barnabe 2014	Canada	Prospective cohort	1116 (826)	54.4 (13.6)	INF, ETA, ADA, CTZ, GOM	30	DAS28 ≤ 2.6	Gender, BMI, RF smoking history
Biggioggero 2019	Italy	Retrospective cohort	346 (282)	53.4 (13)	ETA, ADA	12	DAS28 ≤ 2.6	Age, gender, smoking, disease duration
Canhao 2012	USA	Retrospective cohort	617 (544)	42.2 (12.4)	INF, ETA, ADA	12	DAS28 ≤ 2.6	Age, gender, smoking, disease duration
Collins 2020	USA	Prospective cohort	380 (316)	53.0 (12)	Tocilizumab	6	SDAI ≤ 2.8	Age, gender, disease activity, disease duration
Flouri 2014	Greece	Prospective cohort	1208 (1007)	58 (17)	INF, ETA, ADA	12	DAS28 ≤ 2.6	Gender
Hamann 2019	UK	Retrospective cohort	14,436 (10,971)	56.0 (12.3)	INF, ETA, ADA, CTZ	6	DAS28 ≤ 2.6	Age, gender, smoking, disease activity
Hyrich 2006	UK	Prospective cohort	1,267 (1100)	56 (12)	ETA	6	DAS28 ≤ 2.6	Age, gender, smoking, disease activity, disease duration
Hyrich 2006	UK	Prospective cohort	1612 (1387)	55 (12)	INF	6	DAS28 ≤ 2.6	Age, gender, smoking, disease activity, disease duration
Kawashiri 2021	Japan	Retrospective cohort	125 (90)	59 (18)	INF, ETA, ADA	12	SDAI ≤ 3.3	Use of steroids, ACPA, swollen joint count
Kida 2020	Japan	Prospective cohort	554 (441)	67.8 (12.4)	ABC	13	SDAI ≤ 3.3	Age, gender, ACPA, disease activity, disease duration
Listing 2006	Germany	RCT	818 (627)	55 (12.3)	INF, ETA, ADA, Anakinra	12	DAS28 ≤ 2.6	Age, disease activity
Marie Pers 2013	France	Retrospective cohort	204 (166)	55.2 (13.8)	Tocilizumab	6	DAS28 ≤ 2.6	Age, smoking, disease activity
Murakami 2019	Japan	Prospective cohort	118 (97)	65 (12)	ABC	12	DAS28 ≤ 2.6	Age, gender
Murray 2021	Ireland	Prospective cohort	274 (207)	55 (11.9)	INF, ETA, ADA, RIX	144	DAS28 ≤ 2.6	Age, gender
Nakashima 2020	Japan	Prospective cohort	110 (97)	58.6 (12.8)	Tocilizumab	13	DAS28 ≤ 2.6	MTX use, disease activity
Nourisson 2017	France	Prospective cohort	990 (784)	58.7 (11.6)	ABC, Tocilizumab	12	DAS28 ≤ 2.6	Gender
Rubbert 2021	Austria	Prospective cohort	5462 (4420)	53.3 (12.3)	Tocilizumab	6	SDAI ≤ 3.3	Age, gender, disease activity, disease duration
Tanaka1 2020	Japan	Prospective cohort	159 (NR)	NR	CTZ	13	SDAI ≤ 3.3	Gender, BMI, RF, disease activity
Wang 2019	Taiwan	Prospective cohort	70 (67)	54.1 (10.6)	RIX	24	DAS28 ≤ 2.6	Age, gender, RF, ACPA, disease activity and duration
Yamaguchi 2020	Japan	Prospective cohort	75 (68)	59.7 (10.7)	ADA	6	DAS28 ≤ 2.6	Age, MTX use, disease duration

*INF* infliximab, *ETA* etanercept, *ADA* adalimumab, *CTZ* certolizumab, *GOM* golimumab, *ABC* abatacept, *RIX* rituximab, *DAS28* disease activity score, *SDAI* simple disease activity index, *BMI* body mass index, *RF* rheumatoid factor, *ACPA* anticitrullinated peptide antibody, *MTX* methotrexate

A total of 16,934 patients were included in the 21 studies. Remission criteria was defined as disease activity score (DAS28) of less than or equal to 2.6 [11–23, 26, 28, 30]. Other studies used the simplified disease activity index (SDAI) score of less than or equal to 3.3 to assess for remission [24, 25, 27, 29, 31]. The average follow-up period after staring biologics was around 18 months. Across the 21 studies, the rate of remission was about 53%. Most of the studies reported age, female gender, smoking history, presence of comorbidities, disease duration, and disease activity at the time of diagnosis as predictors of remission. Other studies reported different predictors such as body mass index (BMI), erythrocyte sedimentation rate (ESR), anti-citrullinated protein antibodies (ACPA), and rheumatoid factor (RF). The characteristics of the included studies are described in detail in Table 1. The predictors of remission in RA treated with biologics are summarized in Table 2. We then performed a subgroup meta-analysis for predictors of remission in RA patients treated with TNF-α inhibitors alone as shown in Supplementary Table 3.

**Table 2:** Predictors of all biologics included in the meta-analysis

Risk factor (number of studies)	Effect size (95% CI)	*P* value	*I*^2^	*I*^2^heterogeneity	Egger’s test
Sociodemographic-related risk factors					
Age ≥ 50 (15)	OR 0.98 (0.97, 0.99)	< 0.00001	46%	0.03	0.89
Female gender (16)	OR 0.66 (0.56, 0.77)	< 0.00001	61%	0.0009	0.63
BMI ≥ 30 kg/m^2^ (4)	OR 0.95 (0.91, 0.99)	0.02	65%	0.03	NR
Presence of comorbidities (3)	OR 0.77 (0.51, 1.15)	0.2	79%	0.008	NR
Current of ex-smoker (7)	OR 0.86 (0.75, 0.99)	0.04	67%	0.006	NR
Baseline HAQ score ≥ 2 (9)	OR 0.62 (0.48, 1.27)	< 0.00001	42%	0.09	0.68
Disease-related risk factors					
Disease duration ≥ 10 years (11)	OR 0.99 (0.98, 1.00)	0.18	59%	0.007	0.34
DAS28 at diagnosis ≥ 3.2 (13)	OR 0.90 (0.85, 0.96)	0.0005	88%	< 0.00001	0.65
TJC ≥ 10 (5)	OR 0.99 (0.97, 1.01)	0.33	76%	0.002	NR
SJC ≥ 7 (6)	OR 1.00 (0.95, 1.06)	0.94	79%	0.0002	NR
RF positive (8)	OR 0.99 (0.97, 1.01)	0.24	18%	0.29	NR
ACPA positive (3)	OR 2.52 (1.53, 4.12)	0.0003	0%	0.44	NR
ESR > 20 mm/h (4)	OR 0.99 (0.98, 1.00)	0.009	0%	0.69	NR
Treatment-related risk factors					
Prior or concurrent use of MTX (11)	OR 1.16 (0.9, 1.5)	0.25	85%	< 0.00001	0.33
Prior or concurrent use of steroids (8)	OR 0.97 (0.89, 1.06)	0.48	39%	0.12	NR

### Predictors of remission of RA in patients treated with biologics

A total of fifteen predictors were reported in ≥ 3 studies and included in the systematic review and meta-analysis. The predictors were classified as sociodemographic-related, disease-related, and treatment-related predictors.

### Sociodemographic-related predictors

**Fig. 2:**
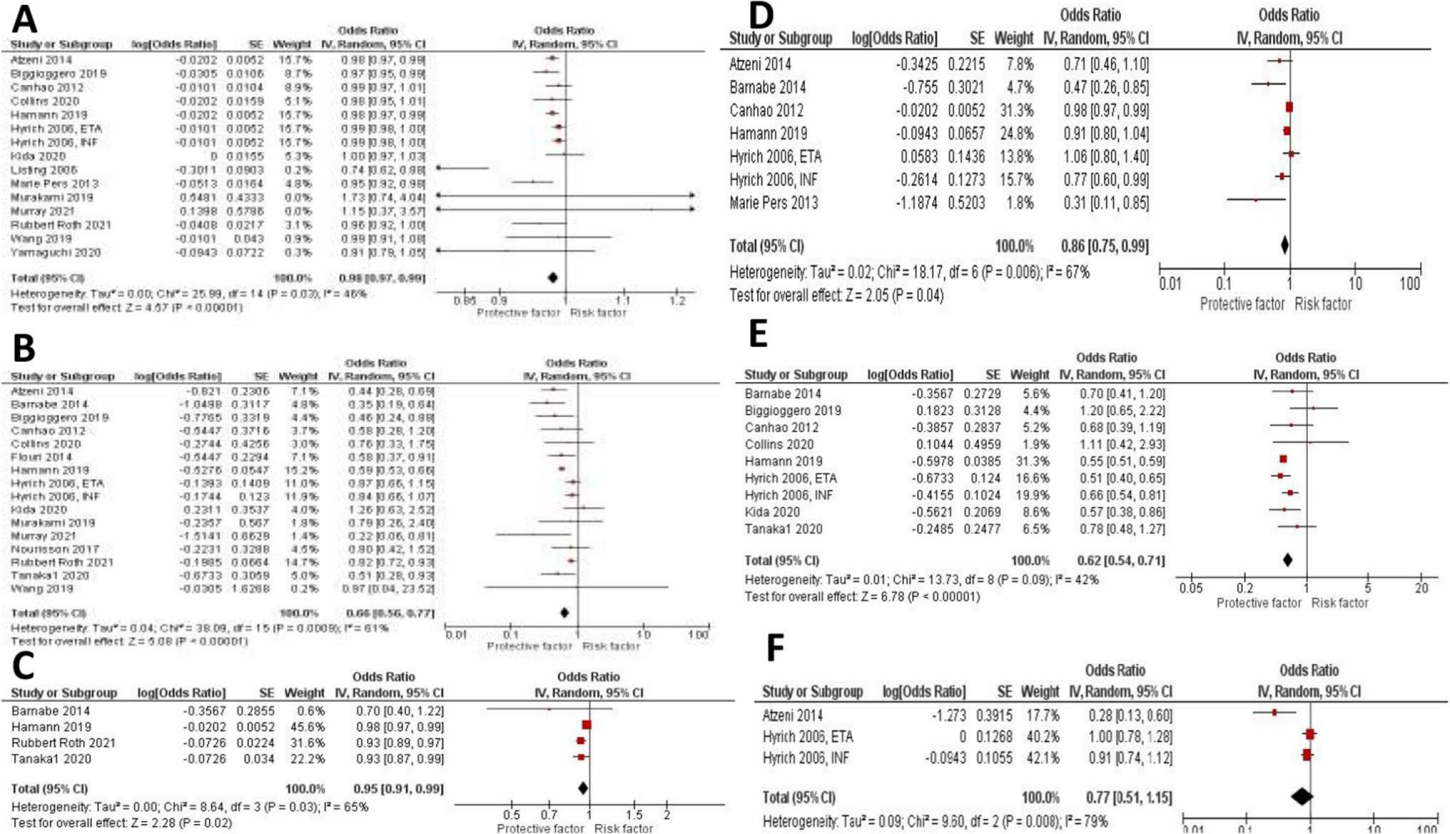
Forest plots of sociodemographic-related predictors of remission of RA in patients treated with biologics: age > 50 years old, female gender, BMI > 30 kg/m.^2^, smoking history, and HAQ score > 2

The effect estimate and forest plot of each predictor are shown in Table 2 and Fig. 2, respectively. We performed meta-analyses for six sociodemographic-related factors including age older than 55 year old (fifteen studies [11, 12, 14, 15, 18, 20–23, 25, 28–31]), female gender (sixteen studies [11, 14–17, 19–25, 27, 30, 31]), obesity defined as BMI ≥ 30 kg/m^2^ (four studies [16, 21, 27, 31]), smoking status defined as current or ex-smoker (seven studies [11, 14–16, 18, 21]), poor baseline functional status defined as Health Assessment Questionnaire (HAQ) of more than two (nine studies [11, 14, 16, 20, 21, 24, 25, 27]), and presence of comorbidities (three studies [11, 15]). Our analysis showed that old age (OR 0.98 (0.97, 0.99), *P* < 0.00001), female gender (OR 0.66 (0.56, 0.77), *P* < 0.00001), BMI > 30 (OR 0.95 (0.91, 0.99), *P* 0.02), smoking history (OR 0.86 (0.75, 0.99), *P* 0.04), and baseline HAQ > 2 (OR 0.62 (0.48, 1.27), *P* < 0.00001) are significantly associated with low rate of remission. Presence of comorbidities, on the other hand, was not associated with significant decrease in remission rate (OR 0.77 (0.51, 1.15), P 0.20). Leave-one-out sensitivity analysis showed consistent results for age, and female gender as shown in Supplementary Fig. 6A and 6B, respectively.

### Disease-related risk factors

**Fig. 3:**
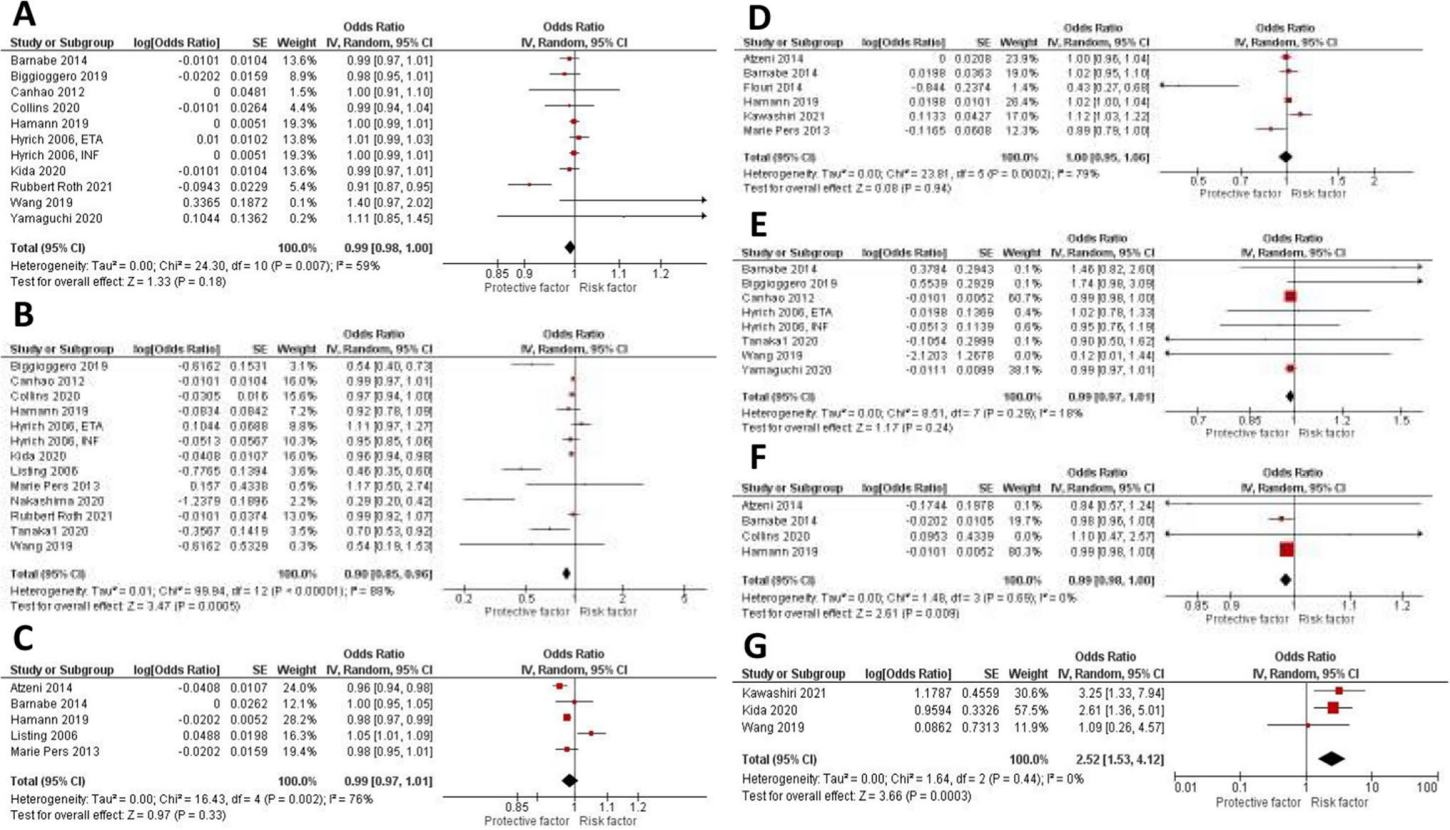
Forest plots of disease-related predictors of remission in RA patients treated with biologics: disease duration ≥ 10 years, DAS28 at time of diagnosis ≥ 3.2, TJC28 ≥ 10, SJC28 ≥ 7, positive RF, positive ACPA, and ESR > 20 mm/h

The effect estimate and forest plot of each predictor are shown in Table 2 and Fig. 3, respectively. We performed meta-analyses for seven disease-related factors including disease duration of more than 10 years (eleven studies [11, 14–16, 20, 21, 23–25, 28, 31]), disease activity score (DAS28) ≥ 3.2 (thirteen studies [11, 12, 14, 18, 20, 21, 23–27, 31]), tender joint count (TJC28) ≥ 10 (five studies [12, 15, 16, 18, 21]), swollen joint count (SJC28) ≥ 7 (six studies [15–18, 21, 29]), positive rheumatoid factor (RF) (eight studies [11, 14, 16, 20, 23, 27, 28]), positive anti-citrullinated protein Antibody (ACPA) (three studies [12, 23, 25]), and elevated erythrocyte sedimentation rate (ESR) > 20 mm/h (four studies [15, 16, 21, 24]). Our analysis showed that high disease activity at the time of diagnosis (OR 0.90 (0.85, 0.96), *P* 0.0005), and elevated ESR (OR 0.99 (0.98, 1.00), P 0.009) are significantly associated with lower remission rate. While disease duration (OR 0.99 (0.98, 1.00), P 0.18), high TJC (OR 0.99 (0.97, 1.01), P 0.33), high SJC (OR 1.00 (0.95, 1.06), P 0.94), and positive RF (OR 0.99 (0.97, 1.01), P 0.24) were all associated with decrease rate of remission, but that was not statistically significant. While positive ACPA was associated with significant increase in remission rate (OR 2.52 (1.53, 4.12), P 0.0003). Leave-one-out sensitivity analysis showed consistent results for disease duration, and disease activity as shown in Supplementary Fig. 6C, and 6D respectively.

### Treatment-related risk factors

**Fig. 4:**
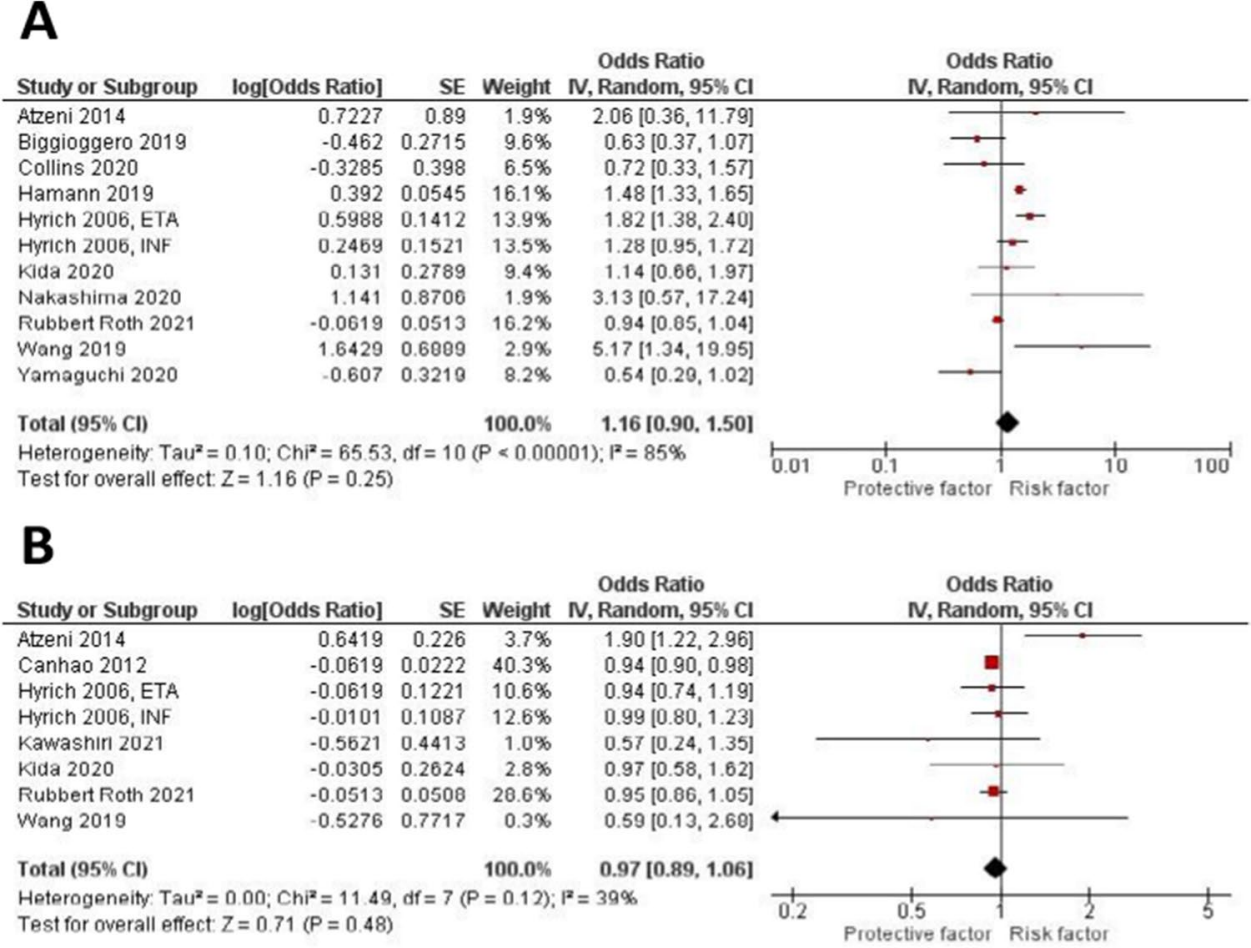
Forest plots of treatment-related predictors of remission in RA patients treated with biologics: prior or concurrent use of MTX, and prior or concurrent use of steroids

The effect estimate and forest plot of each predictor are shown in Table 2 and Fig. 4, respectively. We performed meta-analyses for two treatment-related factors including prior or concurrent use of methotrexate (eleven studies [11, 15, 20, 21, 23–26, 28, 31]), and prior or concurrent use of steroids (eight studies [11, 14, 15, 23, 25, 29, 31]). Our analysis showed that prior or concurrent use of MTX (OR 1.16 (0.9, 1.5), *P* 0.25), and prior or concurrent use of steroids were not associated with significant increase in remission rate (OR 0.97 (0.89, 1.06), *P* 0.48). Consistent results were obtained on leave-one-out sensitivity analysis for MTX use as shown in Supplementary Fig. 6E.

### Subgroup analysis

**Fig. 5:**
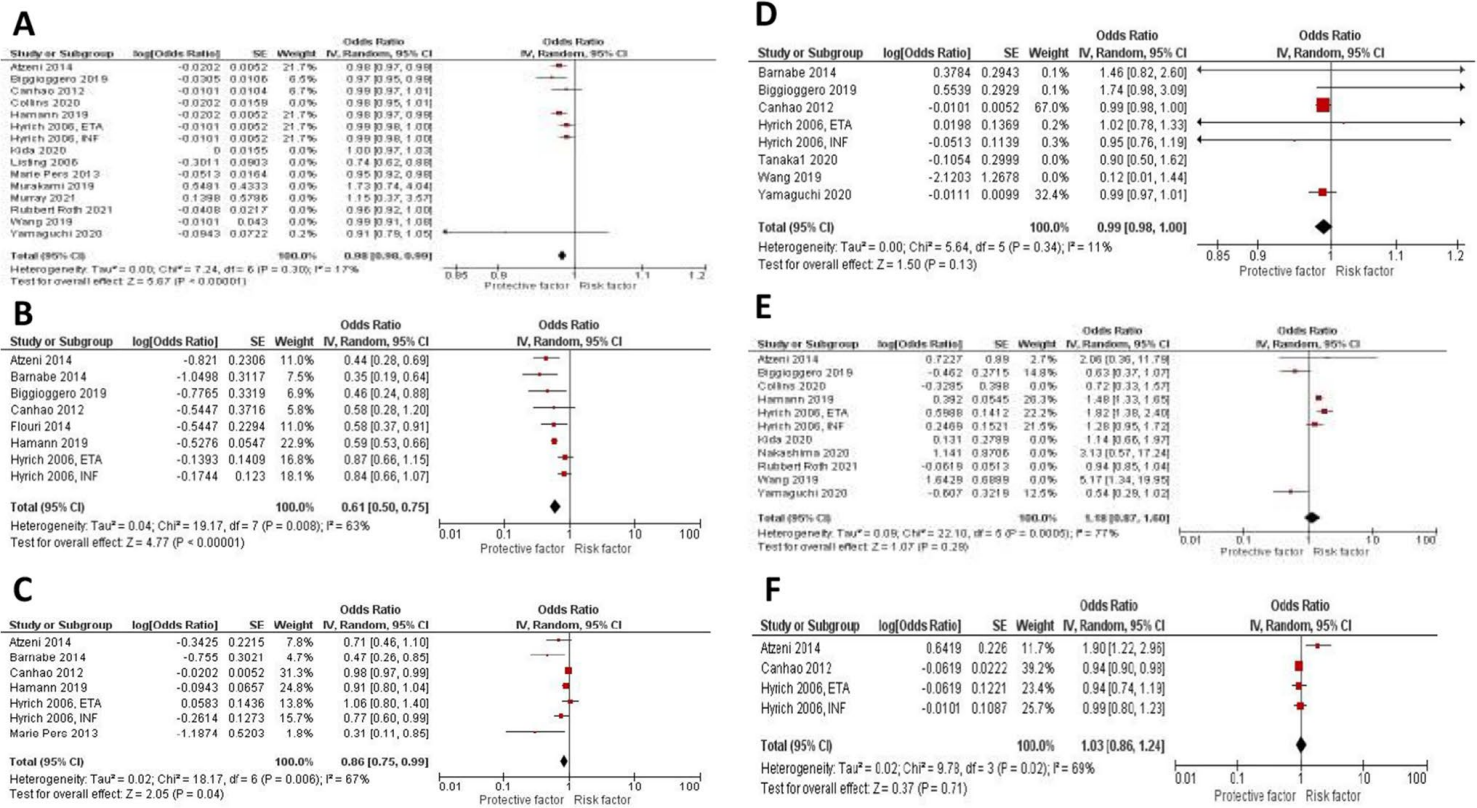
Forest plots for predictors of remission in RA patients treated with TNF-inhibitors alone: age > 50 years old, female gender, smoking history, positive RF, prior or concurrent use of MTX., and prior or concurrent use of steroids

We performed a subgroup analysis to assess for predictors of remission in RA patients receiving tumor necrosis factor alpha inhibitors (TNF-inhibitors). A total of eight studies that included only TNF-inhibitors were used in the subgroup analysis. The effect estimate and forest plot of each predictor are shown in Table 3 and Fig. 5, respectively. Age (OR 0.98 (0.97, 0.99), *P* < 0.00001), Female gender (OR 0.61 (0.50, 0.75), *P* < 0.00001), and smoking history (OR 0.86 (0.75, 0.99), *P* 0.04) were significantly associated with lower remission rate. On the other hand, prior use of MTX (OR 1.18 (0.87, 1.6), *P* 0.29), positive RF (OR 0.99 (0.98, 1.00), *P* 0.13), and prior use of steroids (OR 1.03 (0.86, 1.24), *P* 0.71) were not significantly associated with increasing or decreasing the remission rate.

**Table 3:** Predictors of TNF inhibitors included in the meta-analysis

Risk factor (number of studies)	Effect size (95% CI)	*P* value	*I*^2^	*I*^2^heterogeneity	Egger’s test
Sociodemographic-related risk factors					
Age > = 50 (7)	OR 0.98 (0.97, 0.99)	< 0.00001	17%	0.3	0.01
Female gender (8)	OR 0.61 (0.50, 0.75)	< 0.00001	63%	0.008	NR
Current of ex-smoker (7)	OR 0.86 (0.75, 0.99)	0.04	67%	0.006	NR
Disease-related risk factors					
RF positive (6)	OR 0.99 (0.98, 1.00)	0.13	11%	0.34	NR
Treatment-related risk factors					
Prior or concurrent use of MTX (6)	OR 1.18 (0.87, 1.6)	0.29	77%	< 0.0005	NR
Prior or concurrent use of steroids (4)	OR 1.03 (0.86, 1.24)	0.71	69%	0.02	NR

### Evaluation of publication bias

We used both visual inspection and statistical analysis to assess for publication bias. The funnel plot revealed no publication bias (symmetric) for risk factors reported by ten or more studies, further confirmed by significant Egger’s regression test defined as *P* ≤ 0.01. Funnel plots for predictors of remission in RA patients receiving biologics are shown in Supplementary Fig. 6. Funnel plots were symmetric for age, female gender, disease activity, disease duration, and prior use of MTX, suggesting no publication bias. Moreover, Egger’s test was not statistically significant for these predictors which supports the absence of publication bias. Publication biases of the remaining risk factors could not be performed due to the small number of included studies.

## Discussion

Biologic therapies have successfully revolutionized the management of RA. However, there is a significant proportion of patients who do not respond to the treatment. Identifying the predictors that will affect the treatment response before starting medications with known serious side effects remains challenging. We preformed this systematic review and meta-analysis to investigate the strength of association between different predictors and remission rate in RA patients treated with biologics. In this analysis, 67% of patients achieved complete remission of disease after a follow-up period of 6–12 months. Remission criteria was defined as DAS28 score of less than or equal to 2.6 or SDAI score of less than or equal to 3.3. Old age, female gender, smoking history, obesity, high disease activity at the time of diagnosis, poor functional status, and elevated ESR were associated with lower remission rate. On the other hand, positive ACPA at the time of diagnosis has been associated with higher remission rate. While disease duration, positive RF, prior or concurrent use of steroid, prior or concurrent use of MTX, high TJC, and high SJC score at the time of diagnosis were not significantly associated with lower remission rate. These results were consistent with those treated with TNF-α inhibitors alone.

Many studies supported our findings that women with RA had worse progression of the disease as compared to men despite being on similar treatment [32]. Similar findings have been reported by other studies [33–35]. It has been demonstrated that men and women respond differently to the same treatment due to physiologic differences. Another explanation to our finding is that we used the DAS28 score, which is highly dependent on pain perception, to assess for disease remission. Men may have a higher threshold for reporting joint tenderness which lowers their score. However, we cannot exclude the possibility that men may have a form of the disease that remits more often in comparison with women. Regarding age, our study showed that patient aged > 55 years old were responding poorly to biologics which contradicts the results of other registries that showed no effect of age on response to biologics [11, 36]. Older patients are more likely to have long disease duration which may negatively affect the therapeutic efficacy of biologics. Moreover, elderly patients usually have multiple comorbidities at baseline that make biologic agents potentially more dangerous which results in early discontinuation of these medications.

Obesity, defined as BMI > 30, was found to be a poor predictor of remission in patients receiving biologics. Studies showed that the adipose tissue produces pro-inflammatory cytokines such as TNF-α, and IL-6. The higher fat mass, the higher concentrations of these cytokines which may affect the therapeutic response [37]. Moreover, being a current or former smoker decreases the chances of response to biologics. Smoke acts on both cellular and humoral immunity that leads to a systemic proinflammatory state [38, 39]. Chronic cigarette smoking appears to trigger various morphological, physiological, and enzymatic changes that impairs inflammatory responses [38–40].

Regarding MTX, only 15–20% of our included patients received biologic drugs without prior or concurrent use of MTX. Our analysis showed that MTX prescription at baseline has no significant association with remission. Results were consistent among patients who received TNF- α inhibitors in combination with MTX. Our findings contradict the outcomes of a randomized controlled trial that was conducted in 1998 to investigate the impact of concurrent use of MTX with infliximab in 101 patients with RA [41]. That study showed that MTX has been associated with reduced immunogenicity of infliximab after repeated infusions which helped improve the clinical response. Our results also contradict the outcomes of a network meta-analysis that was conducted in 2019 that also showed that combination therapy of MTX with biologics improved clinical response as compared with biologic monotherapy [42]. Although many studies show that biologic use with MTX improves the clinical outcomes, this should not be considered as a standard of care for different reasons. First, many prescribers require MTX failure before starting biologics. Second, many patients prefer starting MTX prior to biologics because of the cost, and potential side effects. So far, we do not know whether starting biologic treatment rather than MTX improves long-term prognosis given that most of the patients included in the studies were started on MTX prior to biologics. On the other hand, our results should be further investigated by looking at the clinical background of the patients who were started on MTX and those who tried biologics without prior use of MTX. Studies showed that positive RF, younger age at symptom onset, and higher baseline disease activity are associated with higher rates of MTX failure [43]. Further subgroup analysis should be conducted to eliminate the effect of these confounders before making a conclusion.

Currently, there is no biomarker that is known to predict response to biologics in RA patients. Our analysis showed that RF was not significantly associated with poor response to biologics. However, elevated ESR of more than or equal to 20 mm per hour was found to be a significant poor predictor of remission. While patients with positive ACPA showed high remission rate in response to biologics. Several studies reported no relationship between RF or ACPA positivity and the clinical response to tocilizumab treatment [18, 44, 45]. In fact, ACPA positivity has emerged as an important predictor of response to biologics. A post hoc analysis of the AMPLE trial in 2016 initially showed that baseline ACPA positivity was associated with a better response to abatacept and adalimumab [46]. Such association can be explained by the fact that ACPA exert their biological functions by binding to the Fc receptors, expressed particularly by immune cells of the myeloid lineage, and activating the complement system via the classical and alternative pathways [47]. Given that most of the biologics work on inhibiting T-cells, B-cells, and their products of antibodies and inflammatory cytokines, partially explains their relative effectiveness in patients with positive ACPA [48].

Several limitations to our meta-analysis should be mentioned. First, our included studies had inherent bias given their observational nature. Second, there was a significant heterogeneity among the studies that investigated several risk factors such as age, female gender, obesity, smoking, prior use of MTX, baseline functional status, positive RF, and elevated ESR. This heterogeneity could be due to difference in remission criteria, variation in patient demographics, and absence of consistent follow-up period among the studies. Despite the use of the random-effects model to assess for heterogeneity, our results should be interpreted carefully. Third, our study included some methodological limitations that need to be considered while interpreting the results. In our included studies, the patients treated with biologics had long-standing disease and had failed several previous DMARDs. The evaluation of disease remission in these patients using the DAS28 scoring system is tricky given that joint pain and swelling could result from structural and permanent damage due to prolonged disease course. In addition to that, we used ESR value of more than 20 mm/h as a poor predictor of biologics. However, ESR level significantly increases with age, so higher cutoff values should have been considered positive given that most of our patients are older than 40 years old. Moreover, patients were followed-up for an average of 6 months in most of the included studies, and only six out of twenty-one studies had a follow-up period of more than one year which may have affected the response rate to biologics [4]. Finally, some risk factors were excluded given that they were reported in less than three studies such as family history and elevated CRP.

Despite these limitations, our study has several strengths. Up to our knowledge, this is the first meta-analysis that summarizes the available literature and provides a quantitative assessment of different risk factors associated with remission. Moreover, our analysis reported a large cohort of 16,934 patients from twenty-one studies. We also performed sensitivity analysis to the risk factors reported by ten or more studies, and no publication bias was detected in any of them. Finally, our results remained consistent when we preformed subgroup analysis for TNF inhibitors.

In conclusion, RA patients who are females with advanced age, obesity, smoking history, poor functional status, high disease activity, and elevated ESR at the time of diagnosis showed significantly decreased rate of disease remission after receiving biologics. On the other hand, positive ACPA, and prior use of MTX can increase remission rate in these patients. These predictors should be taken into consideration before starting medications with known serious side effects like biologics. Our findings might help develop a clinical prediction model to estimate the rate of remission in RA patients treated with biologics.

Supplementary Fig. 6 Sensitivity analysis for: A, age. B, female gender. C, disease activity. D, disease duration. E, prior use of MTX.

## Supplementary Information

Below is the link to the electronic supplementary material.

Supplementary file1 (PDF 86 KB)

Supplementary file1 (PPTX 271 KB)

## Declarations

The manuscript does not contain clinical studies or patient data.

### Data registration

Data is registered and publicly available at OSF registries. Data including the Review Manager file used for analysis, tables and figures summarizing the included studies, and the predictors that were extracted are all available at OSF registries.
