# Supplementary material for: Predictors of remission in rheumatoid arthritis patients treated with biologics: a systematic review and meta-analysis
Source: Clin Rheumatol. 2022 Aug 16;41(12):3615–27. doi: 10.1007/s10067-022-06307-8 (PMC9652218; doi:10.1007/s10067-022-06307-8)

Supplementary Fig.6 Sensitivity analysis for: A, age. B, female gender. C, disease activity. D, disease duration. E, prior use of MTX

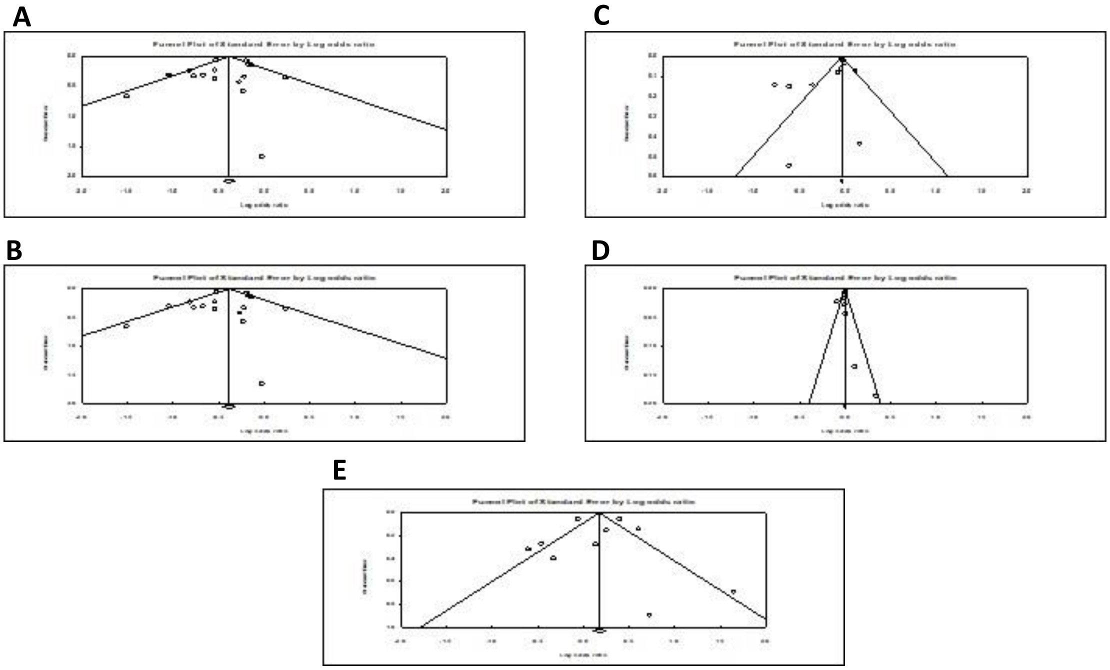

Supplement: Supplementary file 1 — Supplementary file1 (PDF 86 KB) [file 10067_2022_6307_MOESM1_ESM.pdf]
