# Supplementary material for: Predictors of remission in rheumatoid arthritis patients treated with biologics: a systematic review and meta-analysis
Source: Clin Rheumatol. 2022 Aug 16;41(12):3615–27. doi: 10.1007/s10067-022-06307-8 (PMC9652218; doi:10.1007/s10067-022-06307-8)
Supplement: Supplementary file 2 — Supplementary file1 (PPTX 271 KB) [file 10067_2022_6307_MOESM2_ESM.pptx]

## Slide 1
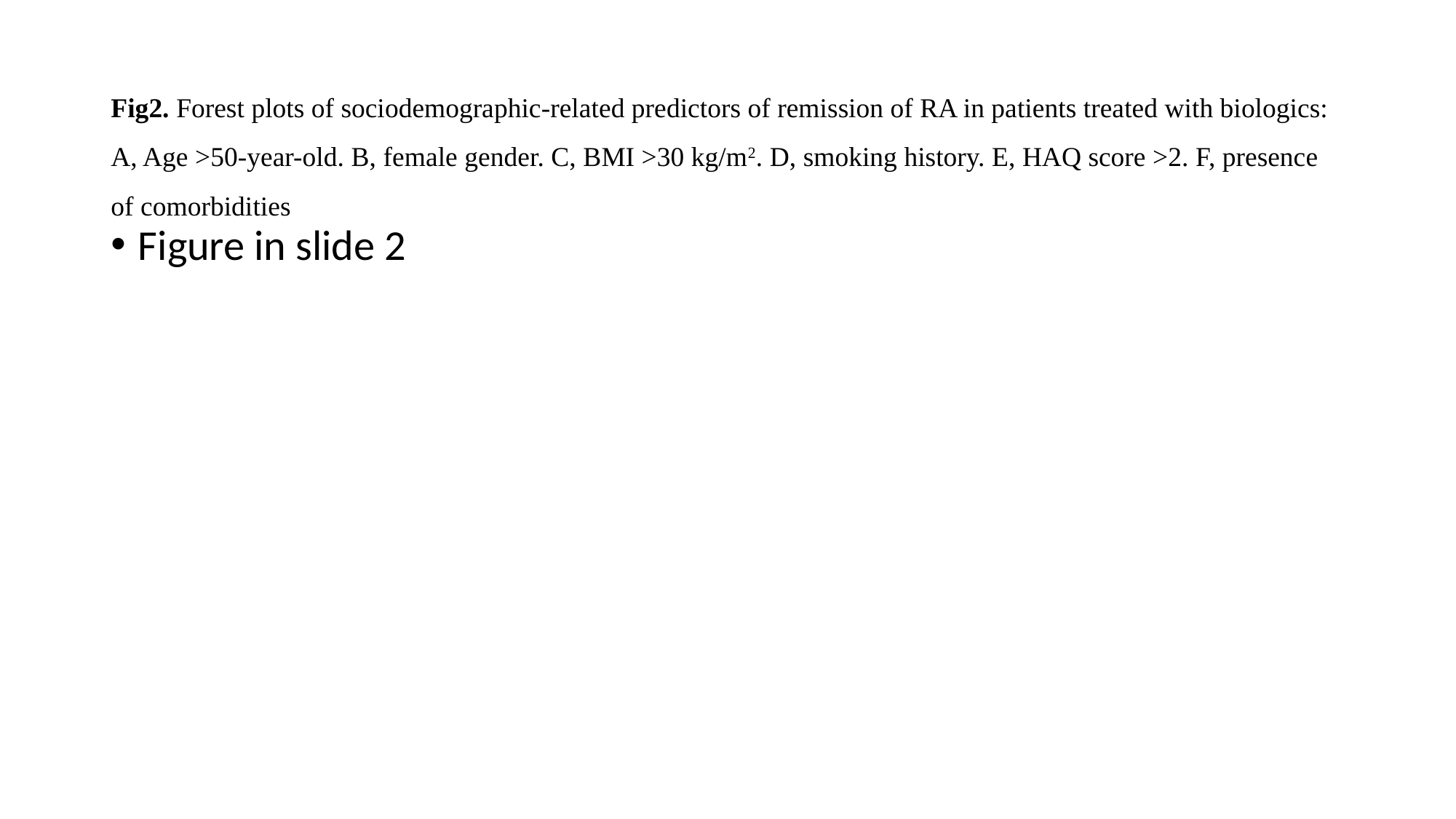

# Fig2. Forest plots of sociodemographic-related predictors of remission of RA in patients treated with biologics: A, Age >50-year-old. B, female gender. C, BMI >30 kg/m2. D, smoking history. E, HAQ score >2. F, presence of comorbidities
Figure in slide 2

## Slide 2
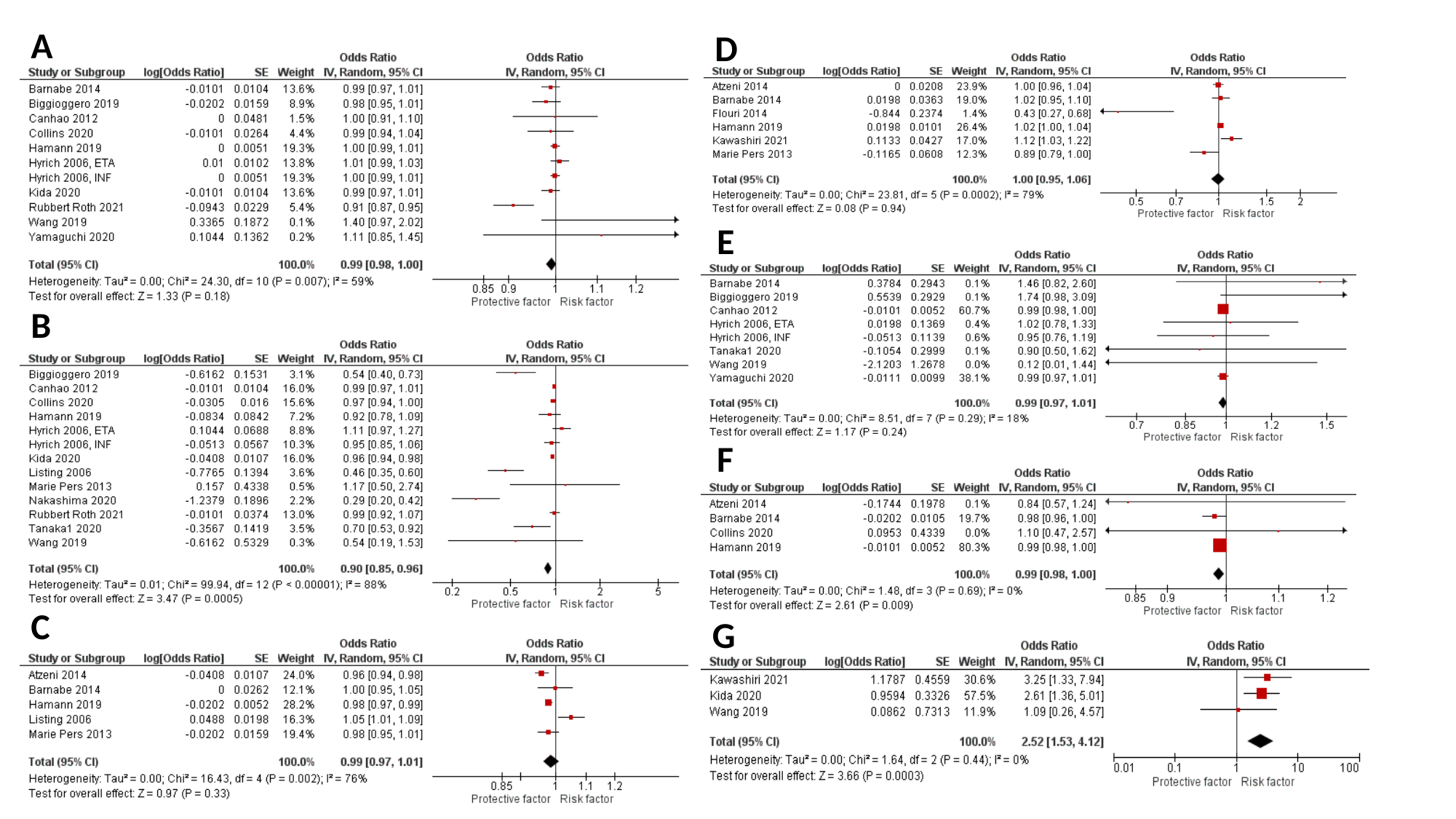

A
D
E
B
F
C
G

## Slide 3
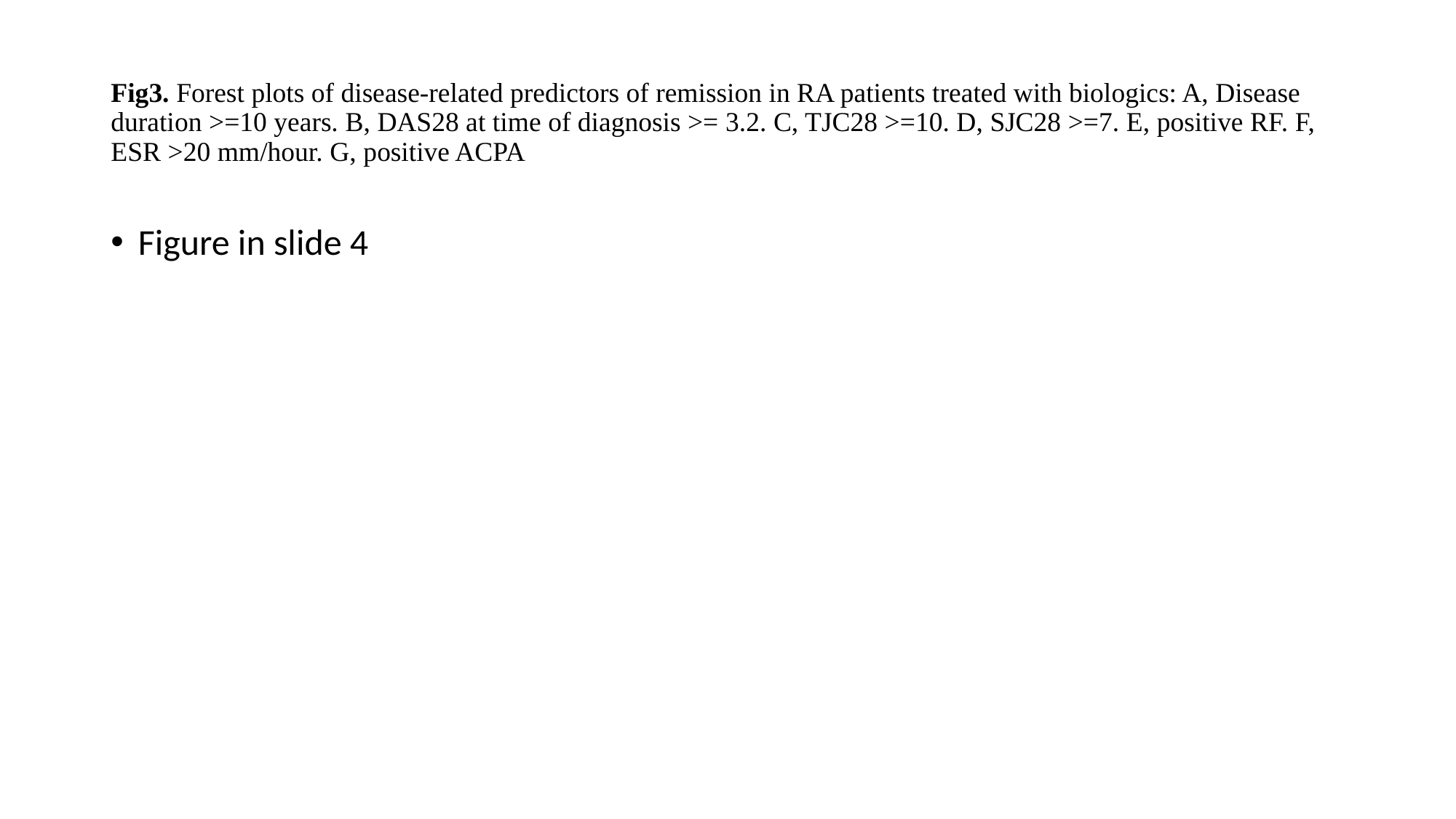

# Fig3. Forest plots of disease-related predictors of remission in RA patients treated with biologics: A, Disease duration >=10 years. B, DAS28 at time of diagnosis >= 3.2. C, TJC28 >=10. D, SJC28 >=7. E, positive RF. F, ESR >20 mm/hour. G, positive ACPA
Figure in slide 4

## Slide 4
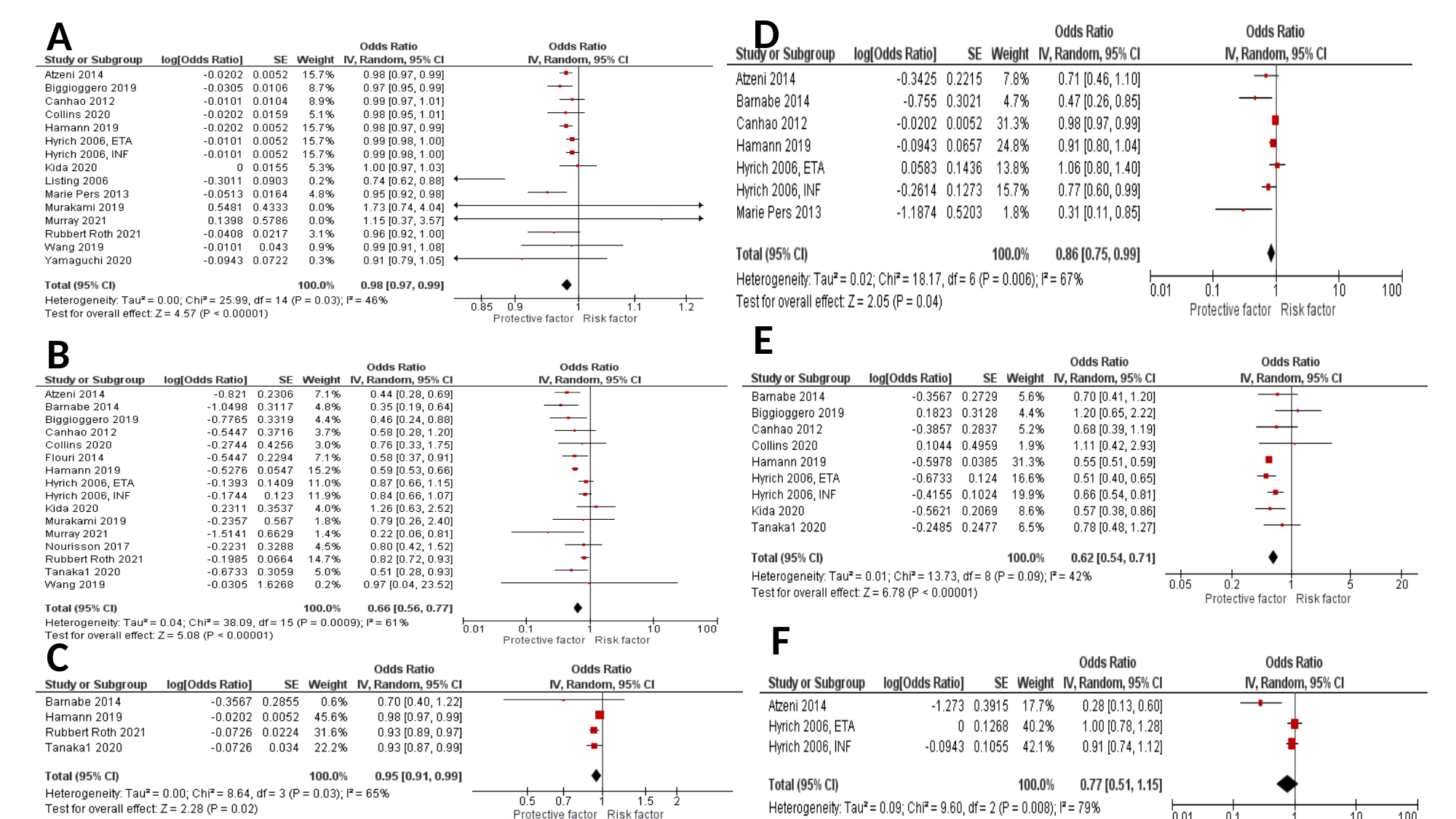

D
A
E
B
F
C

## Slide 5
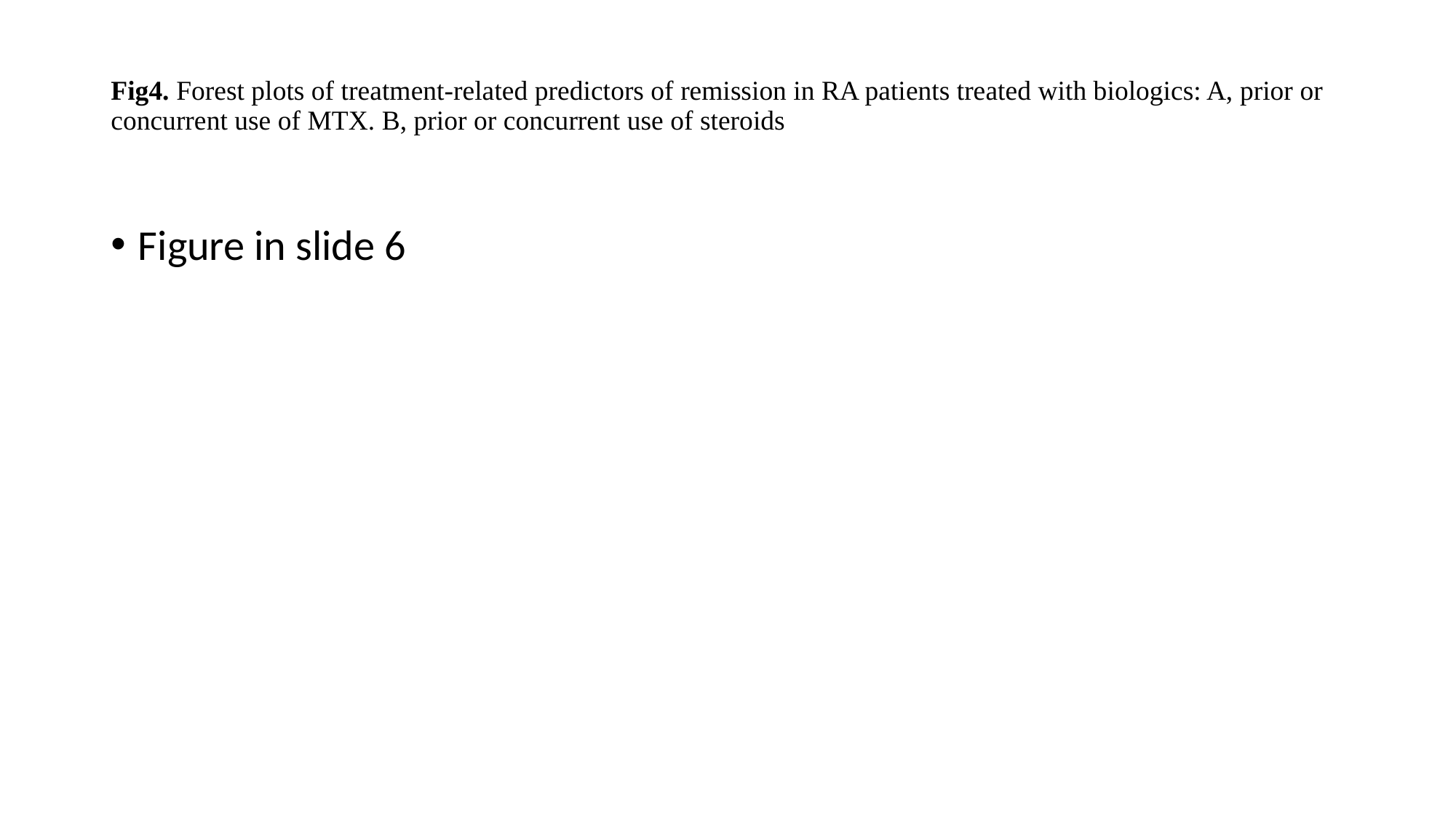

# Fig4. Forest plots of treatment-related predictors of remission in RA patients treated with biologics: A, prior or concurrent use of MTX. B, prior or concurrent use of steroids
Figure in slide 6

## Slide 6
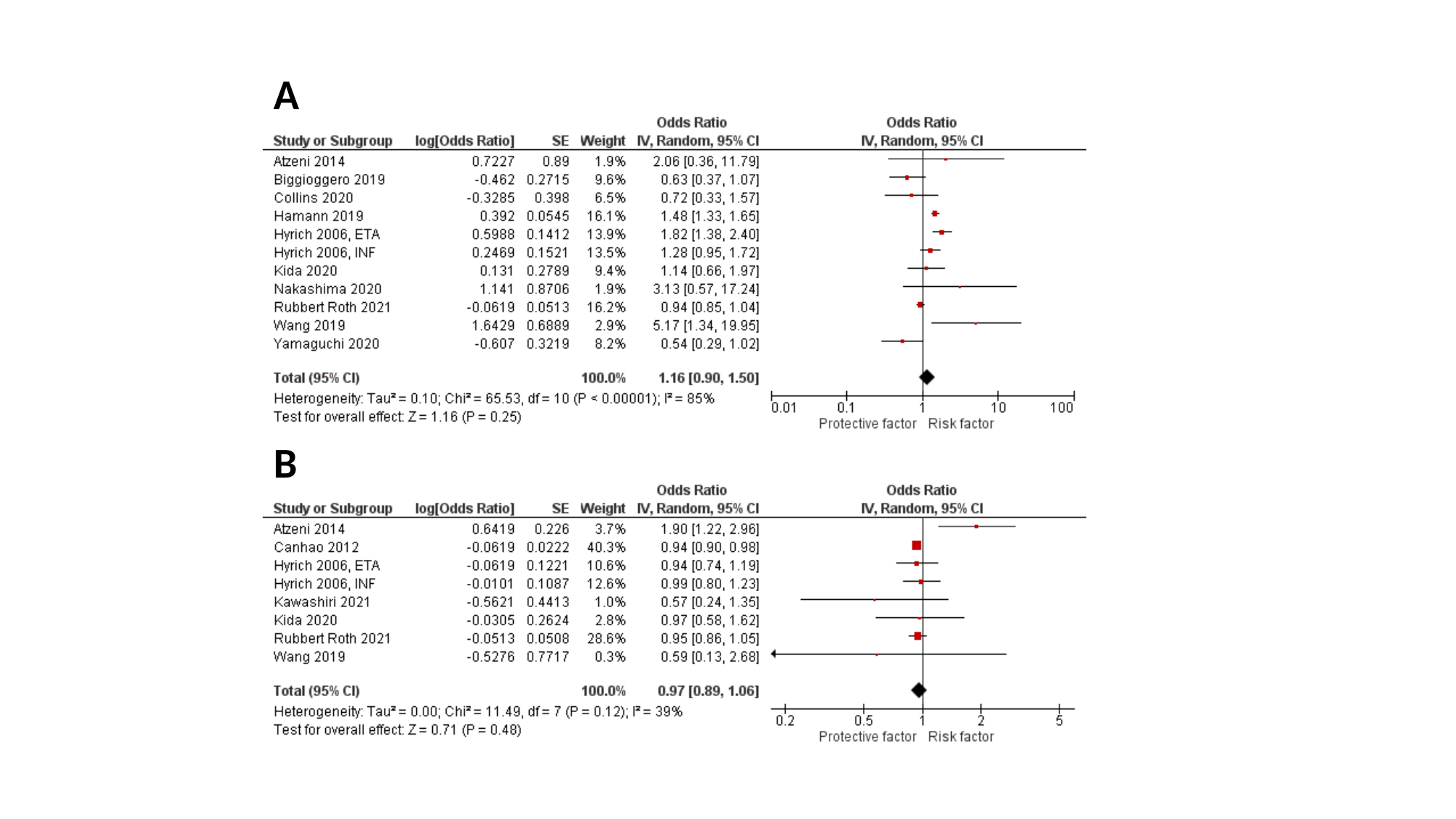

A
B

## Slide 7
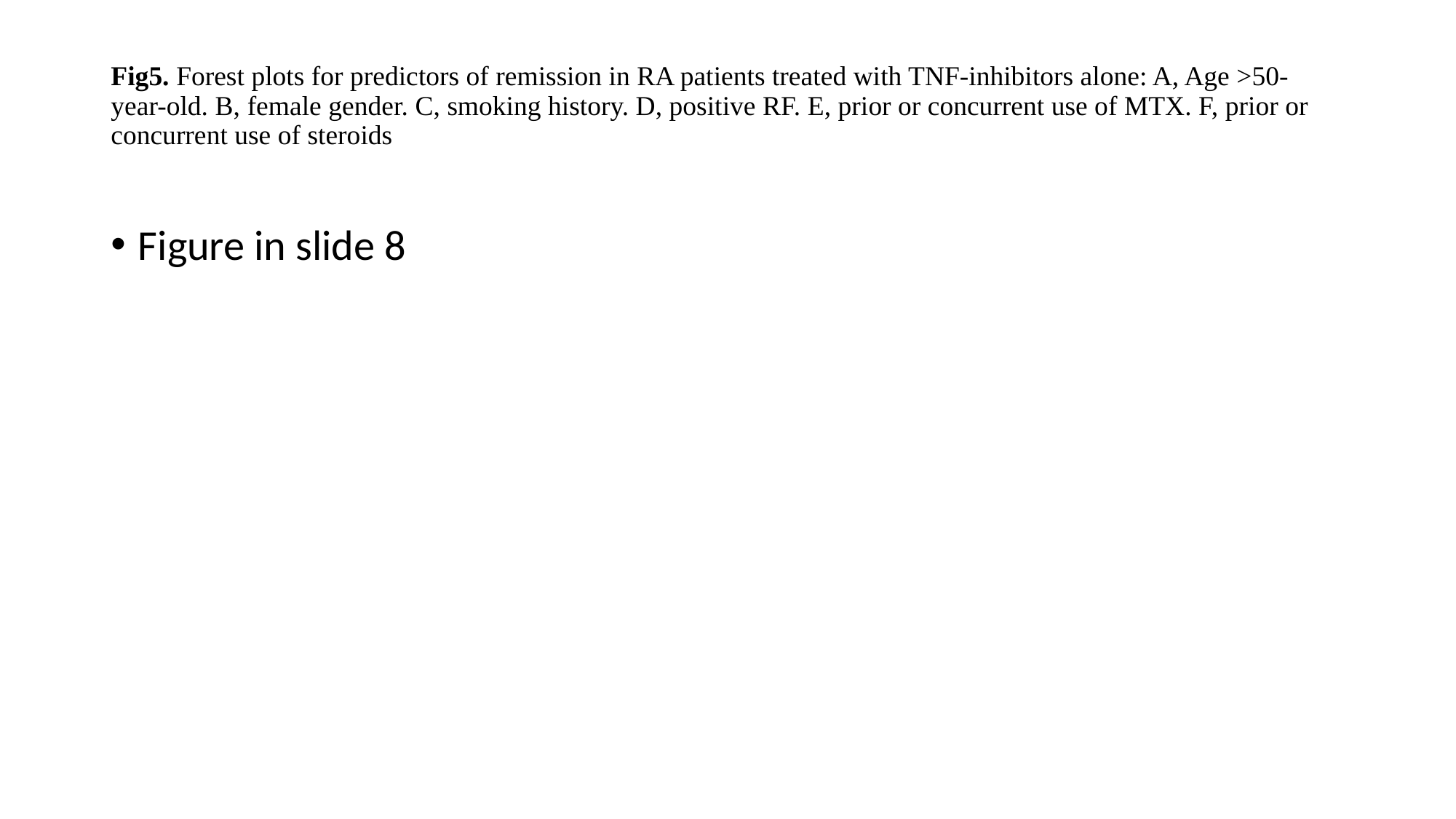

# Fig5. Forest plots for predictors of remission in RA patients treated with TNF-inhibitors alone: A, Age >50-year-old. B, female gender. C, smoking history. D, positive RF. E, prior or concurrent use of MTX. F, prior or concurrent use of steroids
Figure in slide 8

## Slide 8
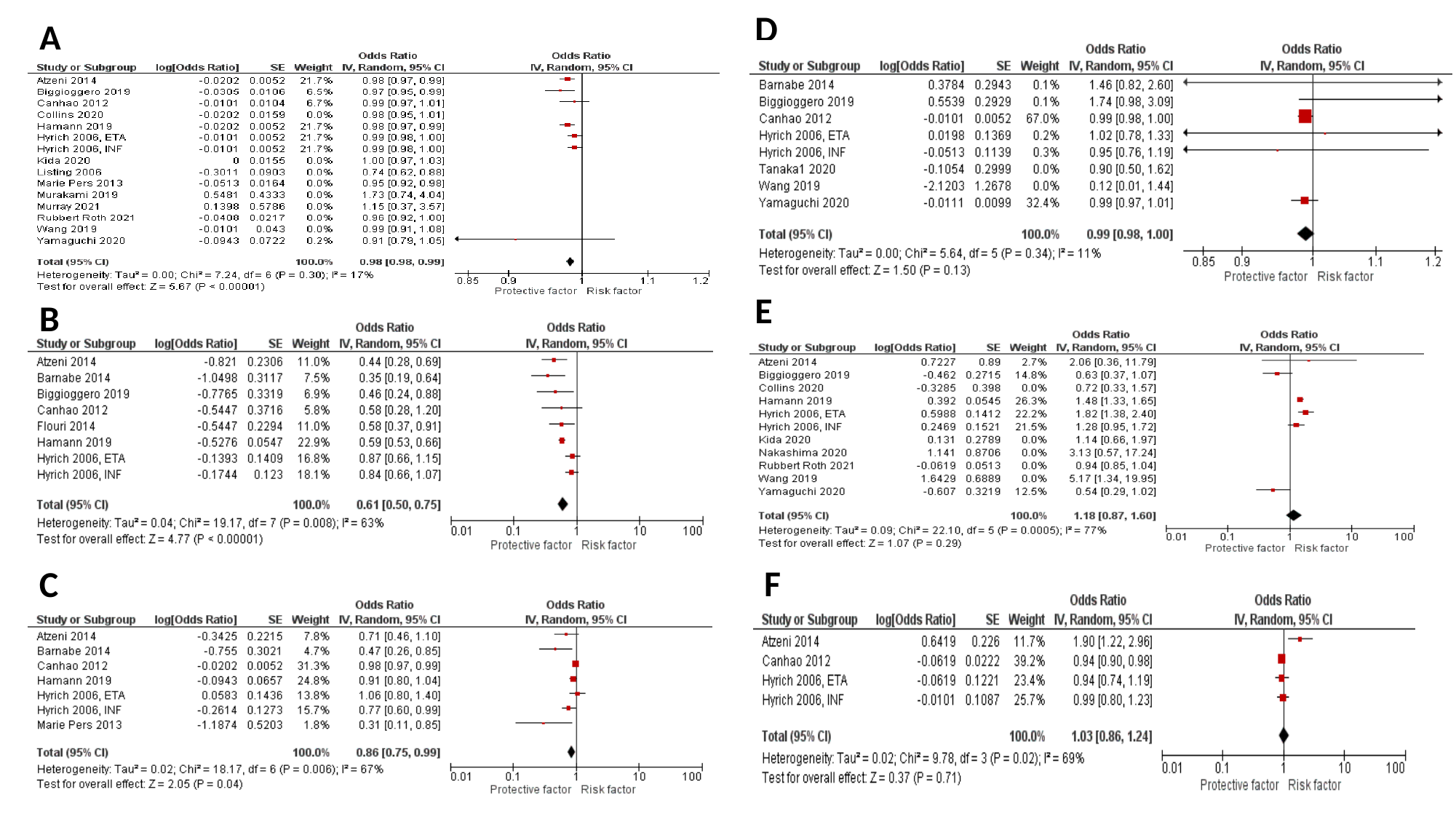

D
A
E
B
F
C

## Slide 9
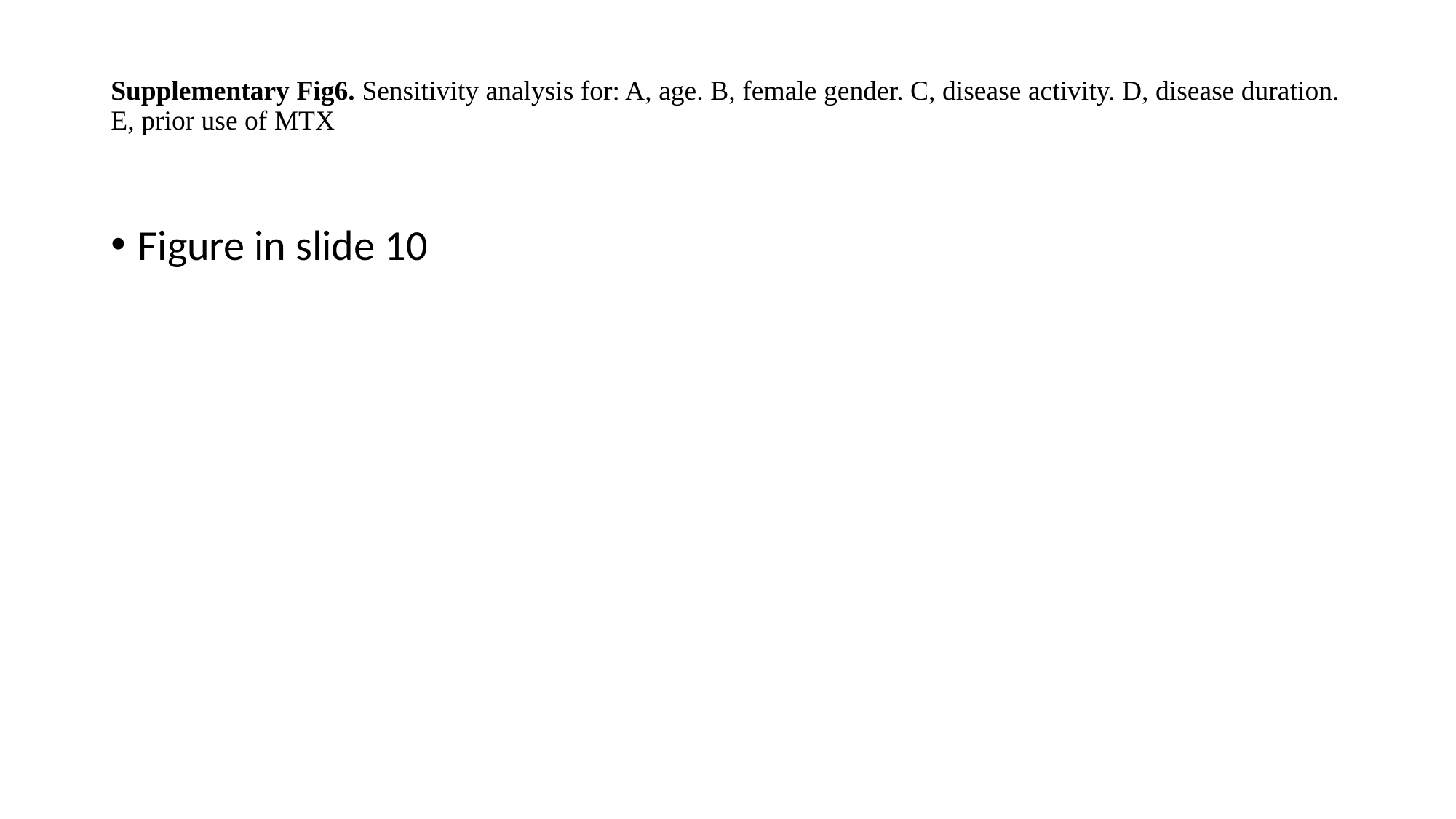

# Supplementary Fig6. Sensitivity analysis for: A, age. B, female gender. C, disease activity. D, disease duration. E, prior use of MTX
Figure in slide 10

## Slide 10
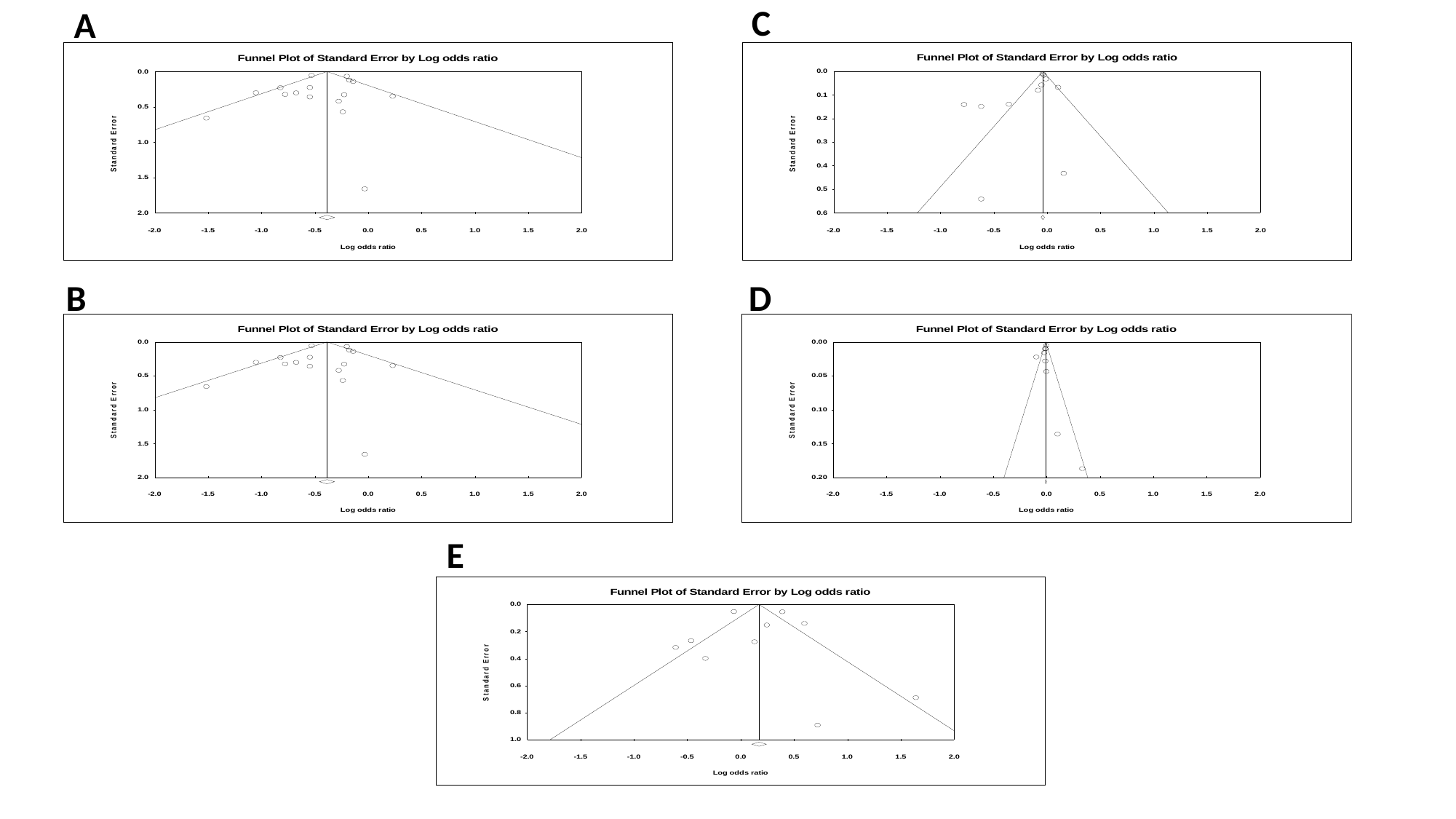

C
A
B
D
E
